# Incidence, Risk Factors, and Outcomes of Neonatal Acute Kidney Injury: Protocol of a Multicentric Prospective Cohort Study [The Indian Iconic Neonatal Kidney Educational Registry]

**DOI:** 10.3389/fped.2021.690559

**Published:** 2021-07-09

**Authors:** Gopal Agrawal, Sanjay Wazir, Sidharth Kumar Sethi, Abhishek Tibrewal, Rohan Dhir, Naveen Bajaj, Naveen Parkash Gupta, Shishir Mirgunde, Jagdish Sahoo, Binesh Balachandran, Kamran Afzal, Anubha Shrivastava, Jyoti Bagla, Sushma Krishnegowda, Ananth Konapur, Kritika Soni, Vamsi Krishna Kolukula, Rupali Jangid, Timothy Bunchman, Rupesh Raina

**Affiliations:** ^1^Department of Neonatology, Cloudnine Hospital, Gurgaon, India; ^2^Department of Paediatric Nephrology, The Medicity Hospital, Kidney Institute, Medanta, Gurgaon, India; ^3^Akron's Children Hospital, Akron, OH, United States; ^4^Department of Neonatology, Deep Hospital, Ludhiana, India; ^5^Department of Neonatology, Madhukar Rainbow Children's Hospital, New Delhi, India; ^6^Department of Paediatrics, Government Medical College, Miraj, India; ^7^Department of Neonatology, Institute of Medical Sciences and SUM Hospital, Bhubaneswar, India; ^8^Department of Neonatology, Aster Mims Hospital, Kottakkal, India; ^9^Department of Paediatrics, Jawaharlal Nehru Medical College, Aligarh Muslim University, Aligarh, India; ^10^Department of Paediatrics, Moti Lal Nehru Medical College, Prayagraj, India; ^11^Department of Paediatrics, ESI Post Graduate Institute of Medical Science Research, New Delhi, India; ^12^Department of Paediatrics, JSS Hospital, JSS Academy of Higher Education and Research, Mysuru, India; ^13^Department of Paediatrics, Kalinga Institute of Medical Sciences Hospital, Kurnool, India; ^14^THB, Sekhmet Technologies Pvt Ltd., Gurgaon, India; ^15^Children's Hospital of Richmond at Virginia Commonwealth University, Richmond, VA, United States

**Keywords:** acute kidney injury, neonatal AKI, urine output, creatinine, KDIGO

## Abstract

**Background:** Acute kidney injury (AKI) is a significant problem in neonates, but the evidence is sparse. Neonatal AKI is an independent risk factor for increased mortality and prolonged hospital stay. There are stark differences in the epidemiology of AKI in neonates amongst the developing and the developed world. Increased prevalence of neonatal sepsis, lack of awareness about neonatal AKI and poor access to pediatric nephrologists add to the improper management of neonatal AKI in the developing countries.

**Methods:** This study is a multicentric, national, prospective cohort study [The Indian iconic Neonatal Kidney Educational Registry (TINKER)] conducted in level 2–3 NICUs in 11 centers across India. We have enrolled nearly 2,000 neonates over the study period. Neonates (≤ 28 days) who were admitted in NICU and those who received intravenous (IV) fluids for at least 48 h for hydration and/or nutrition have been included. Data collection included: (1) baseline demographics (2) daily physiologic and laboratory parameters (3) discharge data. KDIGO workgroup AKI definition modified for neonates was used for defining AKI. Data entry was carried out by individual participating centers using a web-based database (akiregistry.org). De-identified data has been maintained and handled by the principal investigator (PI). This collaboration plans to disseminate data through peer-reviewed publications and through presentations at educational conferences.

**Conclusions:** The purpose of this study is to create the first prospective neonatal all-cause AKI data repository and describe the incidence of neonatal AKI in NICUs in the country and determine the risk factors as well as the outcomes of such neonates—both short-term and long-term outcomes. This will eventually spur therapeutic advancements, facilitate decipherment of epidemiological trends, risk factors as well as outcomes and identify disparities in management across the nation.

## Introduction

Acute kidney injury (AKI) is a significant problem in neonates, but the evidence is sparse (1–4). Neonates are more vulnerable to AKI than older children owing to their functionally immature kidneys (5). Moreover, the physiological characteristics of neonates render them susceptible to hypo-perfusion, increased activity of plasma renin, and reduced-sodium reabsorption from the kidneys (6). Critically ill neonates are at even higher risk as a result of several additional potential exposures (e.g., sepsis, shock, perinatal asphyxia, exposure to nephrotoxic medications).

Evaluation of AKI in neonates possesses specific challenges due to the variation in serum creatinine level evolving in the first few weeks of life; creatinine being affected by gestational age, postnatal age and maternal value of creatinine; and tubular immaturity in neonates (7). Standardization of definition of AKI in neonates has improved our understanding (8). Consensus endorsement of Kidney Disease: Improving Global Outcomes (KDIGO) AKI definition has been instrumental in bringing uniformity in research and clinical trials in neonatal AKI (9).

Multiple studies suggest that the incidence of neonatal AKI varies from 18 to 70% (6, 10–16). Multiple risk factors affect the incidence and the overall outcomes in neonates with AKI. Conditions like perinatal asphyxia, neonatal sepsis, patent ductus arteriosus (PDA) and necrotizing enterocolitis (NEC) are known to be associated with the risk of AKI (17). The use of various drugs [e.g., non-steroidal anti-inflammatory drugs (NSAIDs)] during pregnancy and postnatal life have also been reported as additional risk factors for AKI (18). AKI is associated with increased morbidity and prolonged stay in neonatal intensive care unit (NICU), and is an independent predictor of survival (4, 6, 10–16). Recently, the epidemiology of AKI in critically ill neonates was meticulously studied in a multicentric AWAKEN (Assessment of the worldwide AKI epidemiology in neonates) study and later the same group published risk factors specific to various subgroups (10–13). Prevalence of neonatal AKI varies among high-risk subgroups like preterm neonates (6), very low birth weight (14), neonates with congenital diaphragmatic hernia on extra-corporeal membrane oxygenation (ECMO) (15) and those with perinatal asphyxia (16). AWAKEN study has concluded that neonatal AKI is an independent risk factor for increased mortality and prolonged NICU stay (10). This was the first study to systematically conclude that urine output can be a significant variable in the diagnosis of AKI in neonates. The biggest limitation of this study was its retrospective design. The authors of the AWAKEN study had advised for a similar prospective multi-centric study in future. Moreover, long-term outcomes were not studied by the AWAKEN group (10).

There are stark differences in the epidemiology of AKI in neonates amongst the developing and the developed world. Increased prevalence of neonatal sepsis, lack of awareness about neonatal AKI and poor access to pediatric nephrologists add to the improper management of neonatal AKI in the developing countries. The main basis of treatment of AKI in neonates is prevention, vigilant monitoring and early detection (4). It is therefore essential to build a common platform to generate and share relevant information in order to effectively tackle this menace. Therefore, we propose a multicentric prospective cohort study (monitored by neonatologists as well as pediatric nephrologists) across different clinical settings in India to advance the understanding of AKI, by carefully monitoring the kidney injury and capturing the high risks factors associated with AKI and the cause of the death in AKI patients as well as the long-term outcomes in such infants.

## Materials and Methods

### Study Design

**Figure 1:**
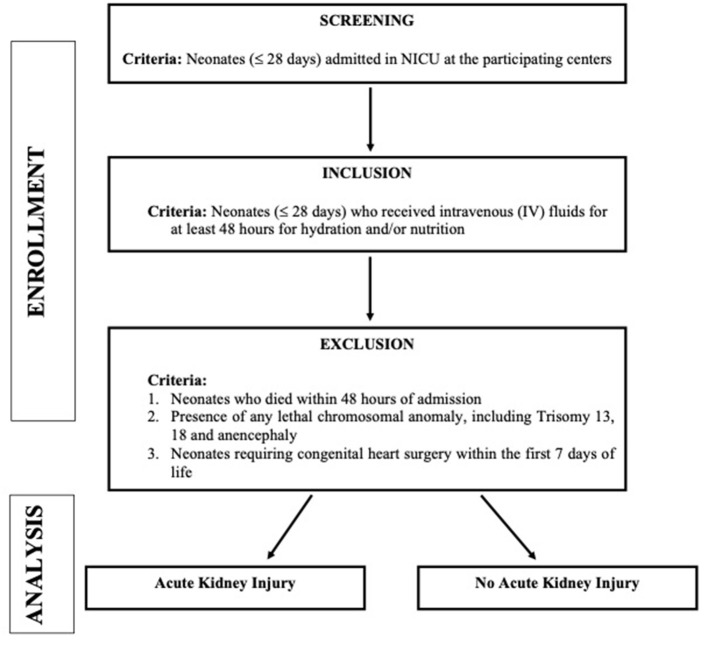
Plan of the study.

This study is a multicentric, national, prospective cohort study [The Indian iconic Neonatal Kidney Educational Registry (TINKER)] conducted in level 2–3 NICUs in 11 centers across India (Table 1 contains list of participating centers along with the name of the leading investigator at the respective center). All the neonates who fulfilled the below mentioned criteria between August 2018 to August 2020 were enrolled. Figure 1 depicts the plan of the study. We have enrolled nearly 2,000 neonates over the study period.

**Table 1:** List of participating centers along with the name of the leading investigator.

**Serial no**	**Name of the hospital**	**Name of the leading investigator**	**State**
1	Cloudnine Hospital, Gurgaon	Sanjay Wazir	Haryana
2	Deep Hospital, Ludhiana	Naveen Bajaj	Punjab
3	Madhukar Rainbow Children's Hospital, New Delhi	Naveen Parkash Gupta	New Delhi
4	Government Medical College, Miraj	Shishir Mirgunde	Maharashtra
5	IMS and SUM Hospital, Bhubaneswar	Jagdish Sahoo	Odisha
6	Aster MIMS Hospital, Kottakkal	Binesh Balachandran	Kerala
7	Jawaharlal Nehru Medical College, Aligarh	Kamran Afzal	Uttar Pradesh
8	MLM Medical College, Prayagraj	Anubha Shrivastava	Uttar Pradesh
9	ESI Post Graduate Institute of Medical Science Research, Basaidarapur	Jyoti Bagla	New Delhi
10	JSS Academy of Higher Education and Research, Mysuru	Sushma Krishnegowda	Karnataka
11	KIMS Hospital, Kurnool	Ananth Konapur	Andhra Pradesh

### Objectives

1. To study the epidemiology of AKI and assess important risk factors of neonatal AKI
2. To determine the association between AKI and other important clinical conditions in such neonates
3. To study the long-term outcomes of neonates with AKI (Chronic kidney disease (CKD), hypertension, proteinuria and growth impairment)

### Inclusion Criteria

1. Neonates (≤ 28 days) who were admitted in NICU; and
2. Those who received intravenous (IV) fluids for at least 48 hours for hydration and/or nutrition

The inclusion criteria have been formulated to include all neonates who are sick and could be at significant risk of AKI. Mildly ill neonates rarely require IV fluids and have a short stay in NICU.

### Exclusion Criteria

1. Neonates who died within 48 h of admission
2. Presence of any lethal chromosomal anomaly, such as Trisomy 13, 18, anencephaly etc.
3. Neonates requiring congenital heart surgery within the first 7 days of life (Because of variability in practices across the centers with regards to management of such neonates and as most of these neonates are transferred to other units post-operatively, we excluded these neonates).

### Data Collection and Entry

A complete clinical history was elicited from all the neonates admitted to the NICU across all centers using paper-based questionnaire/ electronic medical records (EMR) database by the attending doctor. Data entry was carried out by individual participating centers using a web-based database (akiregistry.org). All participating centers used the single standardized online case report form. All participating centers used the single standardized online case record form (See Supplementary Material). Once the inclusion and exclusion criteria were met, the personnel had access to the remainder of the case record form and entered the remaining data.

Serum creatinine was measured daily using enzymatic method till the resolution of AKI episode. Urine output was measured using either diaper or catheter urine collection. Neonates who had renal injury (as per KDIGO classification mentioned below) were managed according to the individual hospital protocol. Blood gas, C-reactive protein (CRP), urine routine and microscopy, urine culture and other blood samples were carried out as per clinical decision.

Data collection included: (1) baseline demographics (2) daily physiologic and laboratory parameters (3) discharge data.

### Baseline Demographics

Demographic data such as maternal variables like age, gravida, parity, chronic and pregnancy-associated conditions, medications, history of peripartum infections, and history of drug intake during the pregnancy (maternal risk factors were identified as per standard ACOG guidelines [American College of Obstetricians and Gynecologists)]; neonatal variables such as gestational age, birth weight, mode and site of delivery, reasons for admission, initial temperature and resuscitation data were documented.

### Data Entry Points

Daily information regarding weight, blood pressure, fluid intake (parenteral+ enteral), total fluid output (urine and others), use of nephrotoxic drugs, and laboratory parameters such as, blood creatinine, urea, sodium, markers of sepsis (blood cultures, urine cultures, and other cultures) were recorded. Above mentioned variables were documented daily during the first week and thereafter first value of the week was noted. We identified significant cardiac disease as hemodynamically significant patent ductus arteriosus (PDA), persistent pulmonary hypertension of the newborn (PPHN), cardiogenic shock and other congenital cardiac disease.

### Discharge Data

The information about disposition status of the infant either discharged home before 120 days or still in the NICU at ≥120 days or transferred to another facility or NICU not in liaison with the national collaboration or history of death before completing 120 days were noted.

### Definition of Acute Kidney Injury

We defined AKI as an increase in serum creatinine of 0·3 mg/dl or more (≥26·5 μmol/L) or 50% or more from the previous lowest value, or a urinary output of <1 ml/kg/h on postnatal days 2–7, according to the KDIGO workgroup AKI definition modified for neonates (Table 2) (9).

**Table 2:** Acute kidney injury KDIGO classification modified for neonates (9, 10).

**Stage**	**Serum creatinine (sCr)**	**Urine output**
0	No change in sCr or rise <0.3 mg/dl	> 1 ml/kg/h
1	sCr rise ≥0.3 mg/dl within 48 h or sCr rise ≥1.5–1.9 × reference sCr within 7 days	> 0.5 ml/kg/h and ≤ 1 ml/kg/h
2	sCr rise ≥2–2.9 × reference sCr	>0.3 ml/kg/h and ≤ 0.5 ml/kg/h
3	sCr rise ≥3 × reference SCr or sCr ≥2.5 mg/dl or receipt of dialysis	≤ 0.3 ml/kg/h

*KDIGO, kidney disease: improving global outcomes*.

### Other Definitions

Definitions of terms in this study were considered in accordance to previous published studies.

### Long Term Outcomes

We plan to follow up the neonates who had AKI during the NICU stay. Growth (weight, height, head circumference), blood pressure, hemoglobin, creatinine, urine albumin: creatinine ratio will be monitored annually till 5 years of age to look at the incidence of CKD, hypertension, proteinuria and growth impairment.

### Statistical Analysis

The variables were entered in an online collaborative database and exported to Microsoft excel. Statistical software (SPSS version 20) will be used for the statistical analyses. Descriptive statistics will be performed to determine differences between neonates with and without AKI. Categorical variables will be summarized as frequencies and percentages and compared by either using χ^2^ or Fisher's exact statistics. Kolmogorov-Smirnov Test will be used to test normality of all continuous variables. For normally distributed variables, means and SDs (Standard deviation) will be calculated while for the other variables, medians and IQRs (Inter-quartile range) will be used. Logistic regression would be used to determine the association between AKI and mortality. Linear regression will be used to determine the association between AKI and length of hospital stay. Logistic and linear regression models will be used to find out independent association between AKI and other clinical outcomes.

Both the groups (AKI vs. Non-AKI) will be compared in terms of baseline demographic variables (age, gender), anthropometry at the time of admission (weight, head circumference, length), clinical parameters (maternal characteristics, drugs prescribed in NICU, complications during NICU stay). The outcome will be analyzed in terms of the parameters at the time of discharge like duration of stay, need for respiratory support, anthropometric parameters at the end of the disposition. Survival analysis (Kaplan-Meier, Cox regression) will be used to compare mortality and other severe morbidities (AKI vs. Non-AKI).

### Ethical Considerations

This study has been approved by the Institutional Ethics Committee, of the respective participating hospitals. Confidentiality of subjects has been strictly maintained and only anonymized and deidentified variables would be taken up for the analysis.

Parents have been adequately informed about the nature of the study. They were provided a detailed information sheet and also received a verbal explanation about the study. Informed consent has been obtained by properly explaining the study to the patients including risks and benefits by a competent doctor. The participation of the enrolled subjects was fully voluntary, and they were allowed to leave the study at any time at their discretion. No sampling, invasive procedure or additional investigation of the neonate were needed for this study. Management of baby was done as per unit protocol, independent of participation in the study.

### Funding

The electronic database (akiregistry.org) was created by self-funding by the investigators and no external funding source was used.

## Discussion

AKI is a pervasive problem with variable etiologies but similar outcomes across all pediatric age groups. AKI is an independent marker for survival, even in neonatal population (4, 6, 10–16). In view of high risk of developing chronic kidney disease (CKD), this vulnerable population needs to be taken care of, and awareness should be developed for the better understanding of the epidemiology of AKI in neonates. Quantification of a problem is a sine qua non for stimulating efforts aimed at reducing its sequelae. It is therefore essential to build a common platform to generate and share relevant information in order to effectively tackle this menace. The purpose of this study was to create the first prospective neonatal all-cause AKI data repository [The Indian iconic Neonatal Kidney Educational Registry (TINKER)] and describe the incidence of neonatal AKI in NICUs in the country and determine the risk factors as well as the outcomes of such neonates—both short-term and long-term outcomes.

This study will describe the incidence of neonatal AKI in the NICUs in the country to uncover the pervasiveness of this seemingly unobtrusive problem (due to lack of precise documentation and diagnostic delays) with obtrusively negative impacts. This will eventually spur therapeutic advancements, facilitate decipherment of epidemiological trends, risk factors as well as outcomes and identify disparities in management.

This study is the first prospective study to use web-based data collection for neonatal AKI. Time required to complete screening and data entry for excluded patients was ~15 min and an average of 1.5 h for included patients. We hope that such multicenter prospective study will provide evidence to create guidelines for pediatricians, neonatologists and pediatric nephrologists who care for such patients. It will also enable them to plan future intervention studies geared toward the reduction of AKI and improved short and long-term renal outcomes, including CKD.

The biggest strength of this study is that this is the first ever initiative carried out in a prospective, multicentric manner with eleven centers all across the country, recruiting new-born babies with a standard criteria. The other strength of this study would be the size of the cohort (nearly 2,000 neonates). Robust number of values of serum creatinine using enzymatic assay (single method) are available for analysis, allowing us to characterize the AKI rates across all the centers. Unlike previous studies, we plan to follow up the neonates with AKI to look at their long term outcomes too. As with many clinical studies, retrospective or prospective, much of the data extraction is done by trained research staff and not by physicians, which might be a limitation of this study too.

## Ethics Statement

The studies involving human participants were reviewed and approved by Institutional Ethics Committee, Cloudnine Hospitals, Bangalore IEC Number: IEC/C9791/0706/18005. Written informed consent to participate in this study was provided by the participants' legal guardian/next of kin.

## Author Contributions

All authors made substantial contributions to conception and design of the study, drafting the article or revising it critically for important intellectual content. All authors gave final approval of the version to be published.

## Conflict of Interest

The authors declare that the research was conducted in the absence of any commercial or financial relationships that could be construed as a potential conflict of interest.

## References

[1] Schneider J Khemani R Grushkin C Bart R. Serum creatinine as stratified in the RIFLE score for acute kidney injury is associated with mortality and length of stay for children in the pediatric intensive care unit. Crit Care Med. (2010) 38:933–9. 10.1097/CCM.0b013e3181cd12e1 20124891

[2] Andreoli SP. Acute renal failure in the newborn. Semin Perinatol. (2004) 28:112–23. 10.1053/j.semperi.2003.11.003 15200250

[3] Akcan-Arikan A Zappitelli M Loftis L Washburn K Jefferson L Goldstein S. Modified RIFLE criteria in critically ill children with acute kidney injury. Kidney Int. (2007) 71:1028–35. 10.1038/sj.ki.5002231 17396113

[4] Charlton JR Boohaker L Askenazi D Brophy PD D'Angio C Fuloria M. Incidence and risk factors of early onset neonatal AKI. Clin J Am Soc Nephrol. (2019) 14:184–95. 10.2215/CJN.03670318 31738181 PMC6390916

[5] Kaur S Jain S Saha A Chawla D Parmar VR Basu S. Evaluation of glomerular and tubular renal function in neonates with birth asphyxia. Ann Trop Paediatr. (2011) 31:129–34. 10.1179/146532811X12925735813922 21575317

[6] Bruel A Rozé J-C Flamant C Simeoni U Roussey-Kesler G Allain-Launay E. Critical serum creatinine values in very preterm newborns. PLoS ONE. (2013) 8:e84892. 10.1371/journal.pone.0084892 24386431 PMC3875547

[7] Walker M Clark R Spitzer A. Elevation in plasma creatinine and renal failure in premature neonates without major anomalies: terminology, occurrence and factors associated with increased risk. J Perinatol. (2011) 31:199–205. 10.1038/jp.2010.82 20651693

[8] Gawadia J Mishra K Kumar M Saikia D. Prediction of severe acute kidney injury using renal angina index in a pediatric intensive care unit. Indian Pediatr. (2019) 56:647–52. 10.1007/s13312-019-1587-2 31477644

[9] Group KDIGOAKIW. KDIGO clinical practice guideline for acute kidney injury. Kidney Int Suppl. (2012) 2:1–138. 10.1038/kisup.2011.32 24571801

[10] Jetton JG Boohaker LJ Sethi SK Wazir S Rohatgi S Soranno DE. Incidence and outcomes of neonatal acute kidney injury (AWAKEN): a multicentre, multinational, observational cohort study. Lancet Child Adolesc Health. (2017) 1:184–94. 10.1016/s2352-4642(17)30069-x 29732396 PMC5933049

[11] Kirkley MJ Boohaker L Griffin R Soranno DE Gien J Askenazi D. Acute kidney injury in neonatal encephalopathy: an evaluation of the AWAKEN database. Pediatr Nephrol. (2019) 34:169–76. 10.1007/s00467-018-4068-2 30155763 PMC6986688

[12] Stoops C Boohaker L Sims B Griffin R Selewski DT Askenazi D. The association of intraventricular hemorrhage and acute kidney injury in premature infants from the Assessment of the Worldwide Acute Kidney Injury Epidemiology in Neonates (AWAKEN) study. Neonatology. (2019) 116:321–30. 10.1159/000501708 31461717 PMC6881521

[13] Starr MC Boohaker L Eldredge LC Menon S Griffin R Mayock D. Acute Kidney Injury is Associated with Poor Lung Outcomes in Infants Born≥ 32 Weeks of Gestational Age. Am J Perinatol. (2020) 37:231–40. 10.1055/s-0039-1698836 31739364 PMC7408289

[14] Carmody JB Swanson JR Rhone ET Charlton JR. Recognition and reporting of AKI in very low birth weight infants. Clin J Am Soc Nephrol. (2014) 9:2036–43. 10.2215/CJN.05190514 25280497 PMC4255405

[15] Gadepalli SK Selewski DT Drongowski RA Mychaliska GB. Acute kidney injury in congenital diaphragmatic hernia requiring extracorporeal life support: an insidious problem. J Pediatr Surg. (2011) 46:630–5. 10.1016/j.jpedsurg.2010.11.031 21496529

[16] Selewski DT Jordan BK Askenazi DJ Dechert RE Sarkar S. Acute kidney injury in asphyxiated newborns treated with therapeutic hypothermia. J Pediatr. (2013) 162:725–9. e721. 10.1016/j.jpeds.2012.10.002 23149172

[17] Cuzzolin L Fanos V Pinna B di Marzio M Perin M Tramontozzi P. Postnatal renal function in preterm newborns: a role of diseases, drugs and therapeutic interventions. Pediatr Nephrol. (2006) 21:931–8. 10.1007/s00467-006-0118-2 16773403

[18] Kaddourah A Basu R Bagshaw S Goldstein SL. Epidemiology of Acute Kidney Injury in Critically Ill Children and Young Adults. N Engl J Med. (2017) 376:11–20. 10.1056/NEJMoa1611391 27959707 PMC5322803

