# Supplementary material for: Incidence, Risk Factors, and Outcomes of Neonatal Acute Kidney Injury: Protocol of a Multicentric Prospective Cohort Study [The Indian Iconic Neonatal Kidney Educational Registry]
Source: Front Pediatr. 2021 Jul 9;9:690559. doi: 10.3389/fped.2021.690559 (PMC8300429; doi:10.3389/fped.2021.690559)
Supplement: Supplementary file 1 [file Table_1.DOCX]

**CASE RECORD FORM**

**Basic details:**

Name: ______________________ Date of birth (dd/mm/yyyy): ___________

Center Name: ______________________ Hospital Registration no. ______________

Father’s name: _____________________ Date of Entry (dd/mm/yyyy): ___________

Age at entry (Hours): _________________ Mobile No.: ________________________

**Baseline Maternal/Antenatal Characteristics:** Tick all that apply

□ Maternal Age (years): ______

□ Gravida: _________. □ Parity: __________

□ Maternal diabetes

□ Maternal pregnancy induced hypertension

□ Maternal infections at or near the time of delivery including bacterial and viral infections

□ IUGR

□ Oligohydramnios (AFI < 5)

□ Polyhydramnios (AFI > 20)

□ **Use of drugs during pregnancy:** Tick all that apply

□ None

□ ACE-inhibitors

- - 1. □ NSAIDs (including Indomethacin, paracetamol)
  1. □ Tobacco
  2. □ Alcohol
  3. □ SSRIs
  4. □ Maternal steroids for fetal maturation

□ Last maternal serum creatinine value (mg/dL): ________ □ Date (dd/mm/yyyy): __________

**Peripartum characteristics:**

□ Site of delivery: Inborn/ Outborn

□ Mode of delivery: Vaginal/Caesarean

□ Any severe peripartum event: Cord prolapse/ Abruption/ Precipitate labor/ None

□ APGAR score at 5 minutes: _________

□ Resuscitation required in the delivery room: Tick all that apply

□ None (aside from drying and stimulation)

□ Supplemental oxygen

□ Positive Pressure Ventilation (PPV)

□ Intubation

□ Chest compressions

□ Epinephrine

□ Normal saline

□ Blood transfusion

□ Not known

**Baseline Neonatal Characteristics:**

□ Gestational Age at birth (Completed weeks): _________ □ Birth weight (g): _________

□ Head circumference at birth (cm): _________ □ Length at birth (cm): _________

□ Gender: Male/ Female/ Ambiguous

□ Date of admission in NICU (dd-mm-yyyy): __________

□ Admission weight (g): _________

□ Reason for admission: _______________________________________________________

**Neonatal Sickness Characteristics:**

□ Duration of stay in NICU (days): ____________

□ Respiratory support required in NICU: Tick all that apply

□ High frequency ventilation

□ Conventional invasive ventilation

□ Noninvasive ventilation

□ CPAP

□ No respiratory support

□ Sepsis (anytime during the NICU stay): Culture positive/ Clinical sepsis/ Probable sepsis/ None

□ Significant cardiac disease: PDA/ VSD/ Duct dependent lesions/ Shock/ PPHN/ Others

□ Necrotizing Enterocolitis (NEC): Yes/ No. If yes, worst stage of NEC (IA/IB/IIA/IIB/IIIA/IIIB)

□ Intraventricular hemorrhage (IVH): Yes/No. If yes, worst stage of IVH (Grade I/ Grade II/ Grade III/ Periventricular echodensity)

□ Jaundice requiring exchange transfusion: Yes/ No

□ Bronchopulmonary Dysplasia (BPD): Yes/ No. If yes, severity of BPD (Mild/Moderate/Severe)

□ Polycythemia requiring partial exchange transfusion: Yes/ No

□ Any surgical intervention: Yes/ No

□ **Physiologic parameters: (Enter values if available or else skip the particular parameter)**

|  | First 12 hours | Day 2 | Day 3 | Day 4 | Day 5 | Day 6 | Day 7 | Day 14 | Day 21 | Day 28 |
| --- | --- | --- | --- | --- | --- | --- | --- | --- | --- | --- |
| Weight (g) |  |  |  |  |  |  |  |  |  |  |
| Blood Pressure (MAP)  Highest  Lowest |  |  |  |  |  |  |  |  |  |  |
|  |  |  |  |  |  |  |  |  |  |  |
| Lowest Temperature (^0^C) |  |  |  |  |  |  |  |  |  |  |
| IV fluid intake (ml/kg/d) |  |  |  |  |  |  |  |  |  |  |
| Enteral fluid intake (ml/kg/d) |  |  |  |  |  |  |  |  |  |  |
| Urine output (ml/kg/h) |  |  |  |  |  |  |  |  |  |  |
| Evidence of fluid overload (Edema, Hepatomegaly etc)  (Mention Yes/ No) |  |  |  |  |  |  |  |  |  |  |
| PaO2/FiO2 ratio |  |  |  |  |  |  |  |  |  |  |
| Multiple Seizure (Yes/No) |  |  |  |  |  |  |  |  |  |  |

*Time in relation to admission in NICU

□ **Lab parameters* (Enter values if available or else skip the particular parameter)**

|  | First 12 hours | Day 2 | Day 3 | Day 4 | Day 5 | Day 6 | Day 7 | Day 14 | Day 21 | Day 28 |
| --- | --- | --- | --- | --- | --- | --- | --- | --- | --- | --- |
| Serum Urea (mg/dl) |  |  |  |  |  |  |  |  |  |  |
| Serum Creatinine (mg/dl) |  |  |  |  |  |  |  |  |  |  |
| Serum Sodium (meq/L)  Highest  Lowest |  |  |  |  |  |  |  |  |  |  |
|  |  |  |  |  |  |  |  |  |  |  |
| Serum Potassium(meq/L)  Highest  Lowest |  |  |  |  |  |  |  |  |  |  |
|  |  |  |  |  |  |  |  |  |  |  |
| Serum albumin (g/dL) |  |  |  |  |  |  |  |  |  |  |
| Lowest Hb (g/L) |  |  |  |  |  |  |  |  |  |  |
| Lowest serum pH |  |  |  |  |  |  |  |  |  |  |

*Include “worst” for the day if more than one value obtained (highest creatinine, highest urea, lowest hemoglobin, highest and lowest sodium, highest and lowest potassium)

**Medications used for the infant:** Tick all that apply

| **Medication** | **Whether used** | **If yes, cumulative duration of use (in days)** |
| --- | --- | --- |
| □ Aminoglycoside | Yes/ No |  |
| □ Vancomycin | Yes/ No |  |
| □ Colistin | Yes/ No |  |
| □ Amphotericin B | Yes/ No |  |
| □ Fluconazole | Yes/ No |  |
| □ Indomethacin | Yes/ No |  |
| □ Ibuprofen | Yes/ No |  |
| □ Furosemide | Yes/ No |  |
| □ ACE inhibitors | Yes/ No |  |
| □ Caffeine | Yes/ No |  |
| **Medication** | **Whether used** | **If yes, cumulative duration of use (in hours)** |
| □ Dopamine | Yes/ No |  |
| □ Dobutamine | Yes/ No |  |
| □ Epinephrine | Yes/ No |  |
| □ Norepinephrine | Yes/ No |  |

□ **Renal diagnoses:**

□ Acute kidney injury or acute renal failure

□ Urinary tract infections (Please include only if there was a positive urine culture)

□ Medullary nephrocalcinosis/ calcifications/ kidney stones (Must be documented on renal ultrasound)

□ Congenital abnormalities of the kidney (Use most severe on Renal USG or Discharge summary)

Please tick all that apply from the list provided.

| Congenital Abnormality |  |  |
| --- | --- | --- |
| □ Hypoplasia/ Dysplasia | **Yes/ No** | If yes, Unilateral/ Bilateral |
| □ Multicystic Dysplastic kidney | **Yes/ No** | If yes, Unilateral/ Bilateral |
| □ Polycystic kidney disease | **Yes/ No** | If yes, Recessive/Dominant/ Unknown |
| □ Renal agenesis | **Yes/ No**  | If yes, Unilateral/ Bilateral |
| □ Renal Ectopia | **Yes/ No** | If yes, Unilateral/ Bilateral |
| □ Hydronephrosis | **Yes/ No** | If yes, mild/moderate/severe |
| □ PUJ |  **Yes/ No** | If yes, Unilateral/ Bilateral  |
| □ Posterior urethral valves | **Yes/ No** | |
| □ Vesicoureteral reflux | **Yes/ No** | If yes, Grade 1/ 2/ 3/ 4/ 5 |
| □ Any other (Specify: ………………………………………………………………………… | | |

**Renal Replacement Therapy:**

□ Modality received: Tick all that apply

□ Peritoneal dialysis

□ Intermittent hemodialysis

□ CRRT

**Disposition:**

□ Discharged home prior to 120 days of age

□ Still in NICU at ≥ 120 days of age

□ Transferred to another facility or NICU not in liaison with the national collaboration

□ Died in hospital at ≤ 120 days

□ Date of disposition (dd-mm-yyyy): ___________

□ Anthropometric measurements at disposition:

□ Weight (g): ___________

□ Length (cm): ___________

□ Head circumference (cm): ___________

**Was the neonate excluded (after enrollment) at any point from the study: □ Yes □ No**

**If yes; mention the reason for exclusion:**

**□ Died within 48 hours of admission**

**□ Lethal chromosomal anomaly, including Trisomy 13, 18 and anencephaly**

**□ Required congenital heart surgery within the first 7 days of life**

**□ Any other: ______________________________________**

**Form completed by:**

Name _________________ Signature ____________________

Date of completion of form (dd/mm/yyyy): ______________________________________________
